# Pertrochanteric osteotomy and distraction femoral neck lengthening for treatment of proximal hip ischemic deformities in children

**DOI:** 10.1007/s11832-016-0711-2

**Published:** 2016-02-18

**Authors:** Mikhail Teplenky, Waleed Mekki

**Affiliations:** Department of Pediatric Orthopedics No. 11, Russian Ilizarov Scientific Center for Restorative Traumatology and Orthopaedics, 6, M.Ulyanova Street, Kurgan, 640014 Russia

**Keywords:** Proximal femoral ischemic deformities, Ilizarov technique, DDH, Avascular necrosis, Coxa vara, Caput valgum, Short femoral neck

## Abstract

**Purpose:**

Proximal femoral ischemic deformities in the pediatric population is a challenging pathological situation. Many surgical techniques have been proposed to treat this problem, with variable reported results. We believe that a C-shaped pertrochanteric osteotomy plus neck lengthening utilizing distraction osteogenesis principles would restore the femoral anatomical ratios between neck, shaft, and the head, and redress the biomechanics of the proximal femur with resultant sufficient containment of the femoral head within the acetabulum.

**Methods:**

We reviewed the results of 19 patients divided into two groups with proximal femoral ischemic deformities. Between 2002 and 2009, preoperative and postoperative clinical examination and radiographs were assessed measuring the neck–shaft angle (NSA), neck–epiphyseal angle (NEA), articulo-trochanteric distance (ATD), lateralization of the greater trochanter (LT), the angle of Wiberg (CEA), index of lateral head displacement by Reimers (IM), and lateral angle of displacement (LDA).

**Results:**

All patients were followed prospectively. Clinical outcome was assessed using Colton’s criteria, which showed average good improvement in function (58.9 %). Radiological indicators were assessed using Kruczynski’s criteria. For group I, the postoperative NSA, NEA, and CEA showed significant change (*p* < 0.01, *p* < 0.001, and *p* < 0.001, respectively). For group II, the postoperative NSA, NEA, and CEA showed significant change (*p* < 0.001, *p* < 0.001, and *p* < 0.001, respectively).

**Conclusion:**

The midterm functional results are favorable for the implementation of pertrochanteric osteotomy and distraction osteogenesis to treat proximal femoral ischemic deformities in the pediatric population.

## Introduction

Ischemic necrosis of the femoral head is a cause of complex deformities of the proximal femur. The structure involved and the degree of the deformity will determine the relationship between the head, neck, and the tip of the greater trochanter [1]. Kalamchi and MacEwen classified patients into four groups with regard to ischemic changes of the physis and ossific nucleus (Table 1). One of the common types of these ischemic deformities is valgus deviation of the epiphysis, femoral neck shortening, hyperplasia of the greater trochanter, and varus deformation of the proximal femur. These changes contribute to subluxation of the femoral head and impair the function of the gluteal muscles, resulting in pain and Trendelenburg lurch [2, 3]. Proximal femoral osteotomies and neck lengthening in childhood have a positive structural effect on the hip joint, as angles, ratios, and biomechanics are restored close to normal. They also help to prevent secondary osteoarthritic changes [4–6]. Many authors have proposed different osteotomy techniques, like double [7, 8], triple [9, 10], and rotational [11, 12] osteotomies. In our patients, neck lengthening is achieved using the principles of distraction transosseous osteosynthesis described by Ilizarov [13, 14] combined with a C-shaped pertrochanteric osteotomy and fixed by an Ilizarov fixator. This study presents the midterm results of implementation of this method.

**Table 1:** Kalamchi and MacEwen classification of femoral AVN

Group	Degree and localization of AVN
I	Ossific nucleus mottling and revascularization
II	Involvement of epiphysis and lateral physis
III	Involvement of epiphysis and central physis
IV	Total involvement of epiphysis and physis

*AVN* avascular necrosis

## Materials and methods

Between May 2002 and December 2009, 19 patients who had received treatment for residual proximal femoral deformities by the Ilizarov technique were followed prospectively. Patients were assigned into two groups according to their age; the average for group I was 6.66 years (range 4–8 years) and that for group II was 11.8 years (range 9–15 years). There were 15 females and four males. The mean follow-up was 59.78 months (range 46–73) for group I and 54.1 months (range 33–103) for group II. Table 2 shows detailed patient demographic data.

**Table 2:** Demographic, diagnostic, and treatment data

Patient	Age (years)	Gender	Side	Pathology	Grade	N/L (cm)	Comp	F/U (months)
Group I								
1	7	F	L	DDH	II	2	None	50
2	6	F	R	DDH	IV	1	None	70
3	5	F	L	As.necr	II	2	None	73
4	8	F	L	DDH	II	1	None	62
5	8	M	R	Sep.cox	IV	1	InfectionI	58
6	4	F	R	DDH	II	2	InfectionI	46
7	6	M	L	Sep.cox	II	3	None	66
8	8	F	R	DDH	IV	2	None	64
9	8	F	R	DDH	II	2	As.necr	49
Group II								
1	12	F	L	DDH	IV	4	None	53
2	11	F	L	DDH	IV	3	InfectionI	46
3	14	F	L	DDH	II	3	None	44
4	10	F	L	DDH	II	2	None	33
5	13	F	L	DDH	IV	3	None	93
6	12	F	R	DDH	IV	4	None	36
7	9	M	L	As.necr	IV	3	Ret.cons	103
8	9	F	R	DDH	II	1	None	46
9	15	M	R	DDH	II	4	InfectionII	38
10	13	F	R	DDH	IV	3	None	49

*F* female; *M* male; *DDH* developmental dysplasia of the hip; *Ret.cons* retarded consolidation; *As.necr* aseptic necrosis; *N/L* neck length gain; *Comp* complication, *F/U* follow-up

Our inclusion criteria were: (1) groups II and IV Kalamchi classification of osteonecrosis; (2) caput valgus neck-epiphysis deformity; (3) mild dysplasia of the acetabulum; (4) mild hip joint osteoarthritic changes; (5) observational period of 3 years or more.

Exclusion criteria were: (1) dysplasia of the acetabulum requiring surgical correction; (2) coxa breva neck-epiphysis deformity; (3) II–III degrees of osteoarthritis.

Etiologically, the two groups comprised 15 joints of aseptic necrosis of the femoral head complicating reduction of developmental dysplasia of the hip (DDH), two joints were dystrophic varus deformity of the femoral neck complicated by aseptic necrosis of the head, and two as a sequelae of septic coxitis. These cases were included with DDH as they conform with our selection criteria.

Clinical history and examination plus plain pelvic radiographs were used for preoperative diagnosis and to follow functional results. Clinical outcome was assessed by Colton’s criteria [15] and radiographic improvement by Kruczynski’s criteria [16].

Studied radiographs of the hip joint were performed in anterior-posterior, lateral, and abduction with internal rotation projections before the operation, during distraction, after apparatus removal, and all throughout the follow-up period. Manual drawing on X-rays was used to assess radiographic parameters as software was introduced later at our center. The following radiographic parameters were calculated: neck–shaft angle (NSA), epiphysis–shaft angle of Alsberg (AA), neck–epiphyseal angle (NEA), articulo-trochanteric distance (ATD), lateralization of the greater trochanter (LT), the angle of Wiberg (CEA), index of lateral head displacement by Reimers (IM), and lateral angle of displacement (LDA) [16]. The NEA is determined as the difference between the NSA and the angle of Alsberg. A value less than 50º was consistent with pathologic valgus deviation of the head. The LT index was calculated by the ratio of the distance from the center of the head to a vertical line drawn through the tip of the greater trochanter to the diameter of the head; a normal value is in the range of 1.15–1.25. Radiographic values were analyzed using the Excel package (2007). The angles and other parameter values were calculated as mean ± standard deviation and the *p*-value of statistical significance was calculated using data dispersion, error, and Student’s *t*-test. A *p*-value less than 0.05 was considered significant. We have interpreted the values of the NSA, NEA, ATD, and CEA detailed in the results. The difference between the preoperative and postoperative values can be seen in Figs. 4 and 5. Details of other parameters and their significant changes are shown in Table 3.

**Table 3:** Radiographic parameters, preoperative, postoperative, and last outcome values

Parameter	Group I, *n* = 9			Group II, *n* = 10		
	Before surgery	After removal of the apparatus	Last outcome	Before surgery	After removal of the apparatus	Last outcome
NSA (°)	95 ± 2.9	121 ± 1.7* *p* < 0.001	108 ± 1.4* *p* < 0.01	98 ± 2.5	129 ± 1.7* *p* < 0.001	121 ± 1.6* *p* < 0.01
NEA (°)	21.5 ± 3.9	64 ± 0.9* *p* < 0.001	61 ± 1.2	23.5 ± 4.3	70 ± 1.6* *p* < 0.001	67.6 ± 1.8
ATD (mm)	(−8.4) ± 2.5	14.5 ± 0.9* *p* < 0.001	8 ± 1.04* *p* < 0.01	(−13) ± 3.1	17.4 ± 0.8* *p* < 0.001	15.4 ± 1.0
LT (ratio)	0.8 ± 0.04	1.04 ± 0.03* *p* < 0.01	0.88 ± 0.08	0.83 ± 0.03	1.08 ± 0.04* *p* < 0.01	1.02 ± 0.03
CEA (°)	3 ± 0.9	19.5 ± 0.8* *p* < 0.001	17.6 ± 1.0	3.8 ± 1.2	21.6 ± 1.0* *p* < 0.001	20.7 ± 1.3
IM (%)	68 ± 2.3	91 ± 2.2* *p* < 0.001	86 ± 2.7	68 ± 2.7	91.5 ± 2.4* *p* < 0.001	88 ± 1.3
LDA (°)	30 ± 1.6	23.6 ± 2.2* *p* < 0.05	25 ± 1.2	30.5 ± 1.6	21.7 ± 0.8* *p* < 0.01	22.6 ± 0.7

* Significant *p*-value

### Surgical technique

**Fig. 1:**
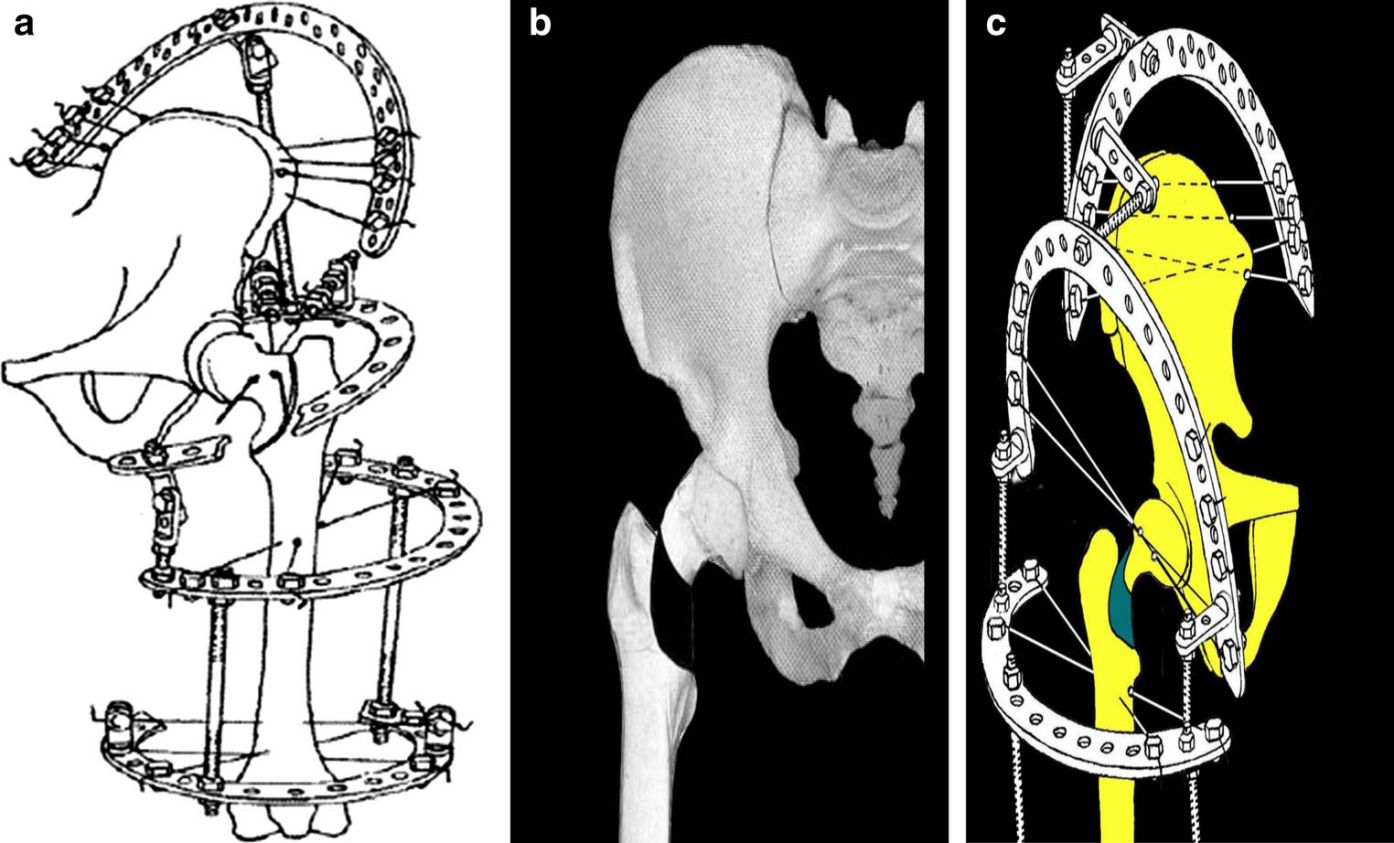
**a**–**c** Schematic representation of the Ilizarov apparatus applied with C-shaped pertrochanteric osteotomy and neck lengthening

**Fig. 2:**
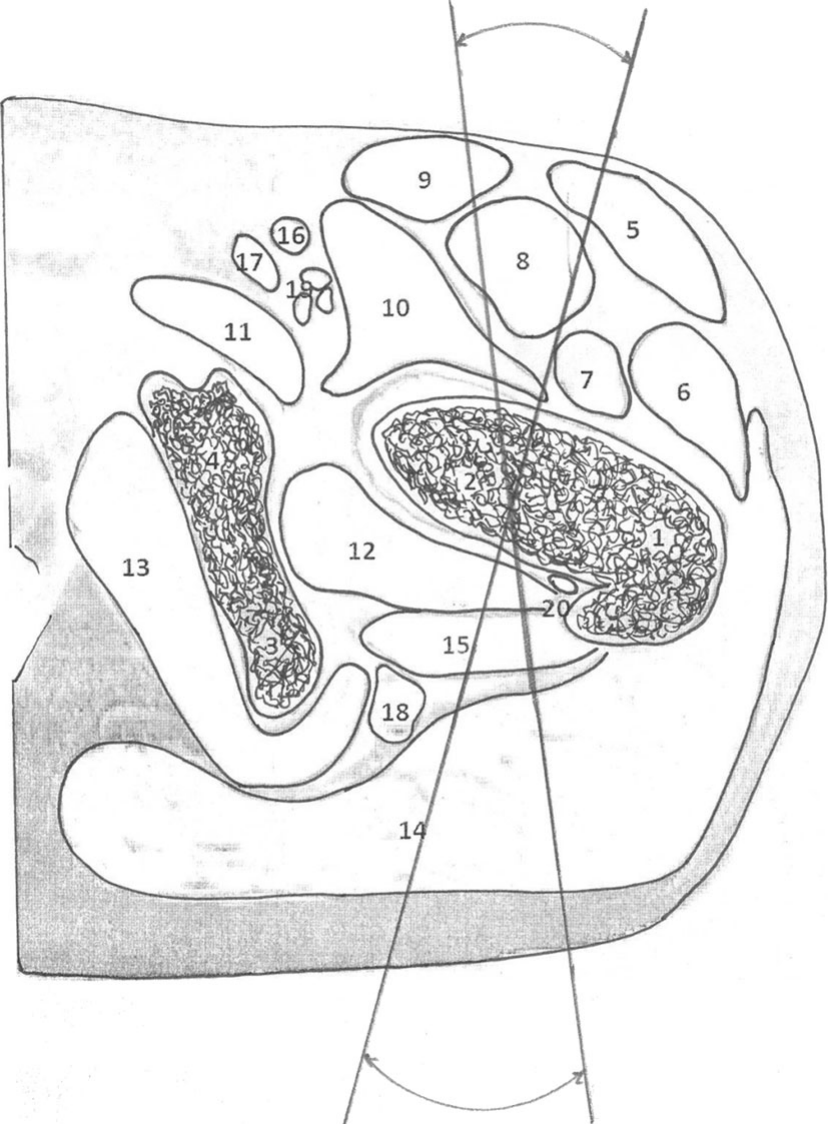
Anatomical illustration of the axial section at the level of the inferior femoral neck. *1* Greater trochanter. *2* Femoral neck. *3* Ischium. *4* Pubis. *5* Tensor fascia lata. *6* Vastus lateralis. *7* Vastus intermedius. *8* Rectus femoris. *9* Sartorius. *10* Iliopsoas. *11* Pectineus. *12* Obturator externus. *13* Obturator internus. *14* Gluteus maximus. *15* Quadratus femoris. *16* Femoral artery. *17* Femoral vein. *18* Sciatic nerve. *19* Femoral nerve. *20* Medial femoral circumflex artery. The angle between the wires is 20°. Note the relation of the wires to the sciatic and femoral nerves and vessels. Insertion closer to the base of the neck is recommended

With image intensifier control and the patient lying supine on the operating table, the affected limb is draped so that the hip area is totally free for wire insertion. We insert four 1.8-mm wires with stop olives through the wing of the ilium. Wire insertion as illustrated in Fig. 1 is safe as far as the wires are passed in the orientation of the iliac wing, which is usually 45° to the operating table. The wires pass through skin, subcutaneous tissues and bone and exit through same structures. Another two olive wires will be passed through the base of the neck to stabilize the proximal part during the distraction phase. The operating surgeon must be mindful of the changing anatomical pattern of different age groups [17] before inserting wires into the neck and, especially for younger patients, the wires should be inserted from the anterior to the posterior direction, with the two wires 20° apart in the horizontal plane (Fig. 2). At this orientation, the wires are far from the sciatic and femoral nerve and vessels. The wires should be of smaller diameter (1.5 mm) and care should be taken not to pass wires either superior or inferior to the neck, as the major blood supply comes from the supero-posterior and, to a lesser extent, from the postero-inferior branches of the medial femoral circumflex artery [17]. One Schanz pin with a diameter not more 2.5 mm can also be used in the neck. Through the lateral part of the middle and lower third of the thigh, we insert additional wires and pins to form two distal levels and connect the rings by rods and hinges to form the four levels of the Ilizarov apparatus (Fig. 1). For the use of pins in the ilium or shaft of the femur, larger diameter pins (4–6 mm) are to be used, depending on the patient size and skeletal maturity. The limb is then positioned in abduction, extension, and internal rotation. With radiographic assessment, we determine the location and direction of the osteotomy and insert multiple k-wires along the line from the greater to the lesser trochanter in a C-shaped manner and with partial disassembly of the frame at the osteotomy site, we make 2–3 longitudinal incisions of 1–1.5 cm on the anterior surface of the hip joint more to the sagittal direction from the trochanteric fossa to the upper edge of the lesser trochanter. A 5-mm osteotome along the weakened line by multiple wires is used to perform the C-shaped osteotomy. The final breach of the osteotomy fragments is produced by rotation of an elevator inserted into the osteotomy site. The correction of the caput valgum deformity of the epiphysis is done at this stage by adducting the whole lower limb, where rotation will happen in the C-shaped axis, this will bring the caput valgum into the corrected position. The high-riding greater trochanter, neck–shaft varus, and short neck will be corrected by gradual distraction postoperatively.

### Postoperative course

**Fig. 3:**
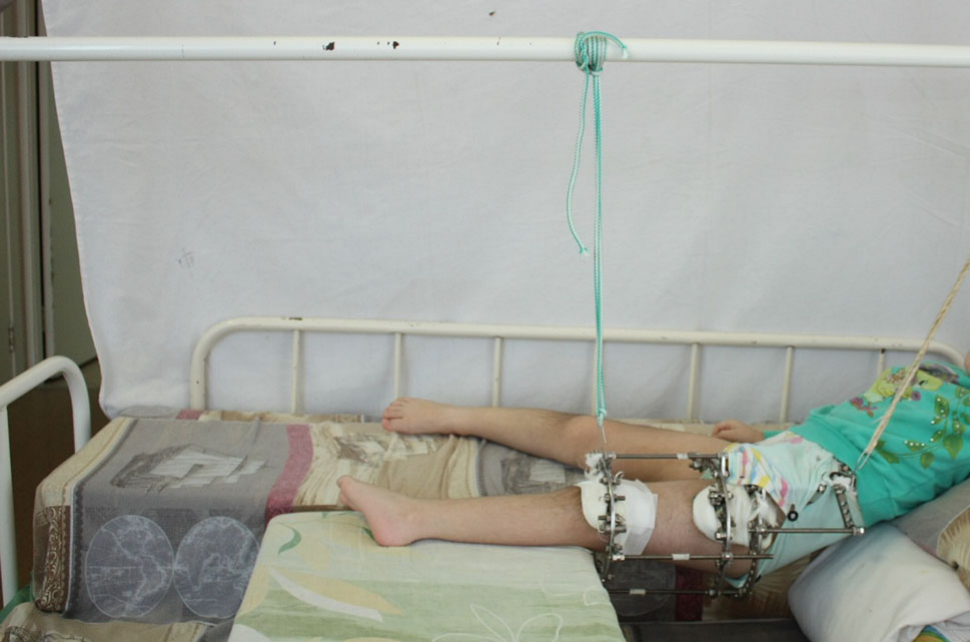
Nursing bed for the Ilizarov apparatus with bolsters arranged to make space for frame accommodation

Dressing gauze with chlorhexidine antiseptic is applied around wires, changed on the second postoperative day, and then regularly every 10 days. Final correction of the deformity is achieved by gradual distraction in the postoperative period by turning the nuts distal to the osteotomy line by a rate of distraction of 1 mm per day (Fig. 1). The length of subsequent fixation in the frame is 2–2.5 months. Patients are nursed in ordinary hospital beds; however, a space is made by bolsters to accommodate the frame (Fig. 3). With adaptation, the patient can rest on ordinary beds with a slight tilt to the contralateral side. The patient can walk supported with only toe touch allowed during the treatment phase in the hospital. Following removal of the frame and discharge, the patient starts passive range of motion (ROM) exercise to the hip and partial weight bearing is allowed for 2–2.5 months. Then, the patient is allowed to do active ROM and to bicycle for another 2–2.5 months. Full loading on the operated limb is permitted in 5–6 months after apparatus removal. These instructions must be communicated in a clear way to the patients or guardians. The follow-up program is after 2, 3, and 6 months. After this critical period is passed, patient visits are set to once yearly or as deemed necessary.

## Results

**Fig. 4:**
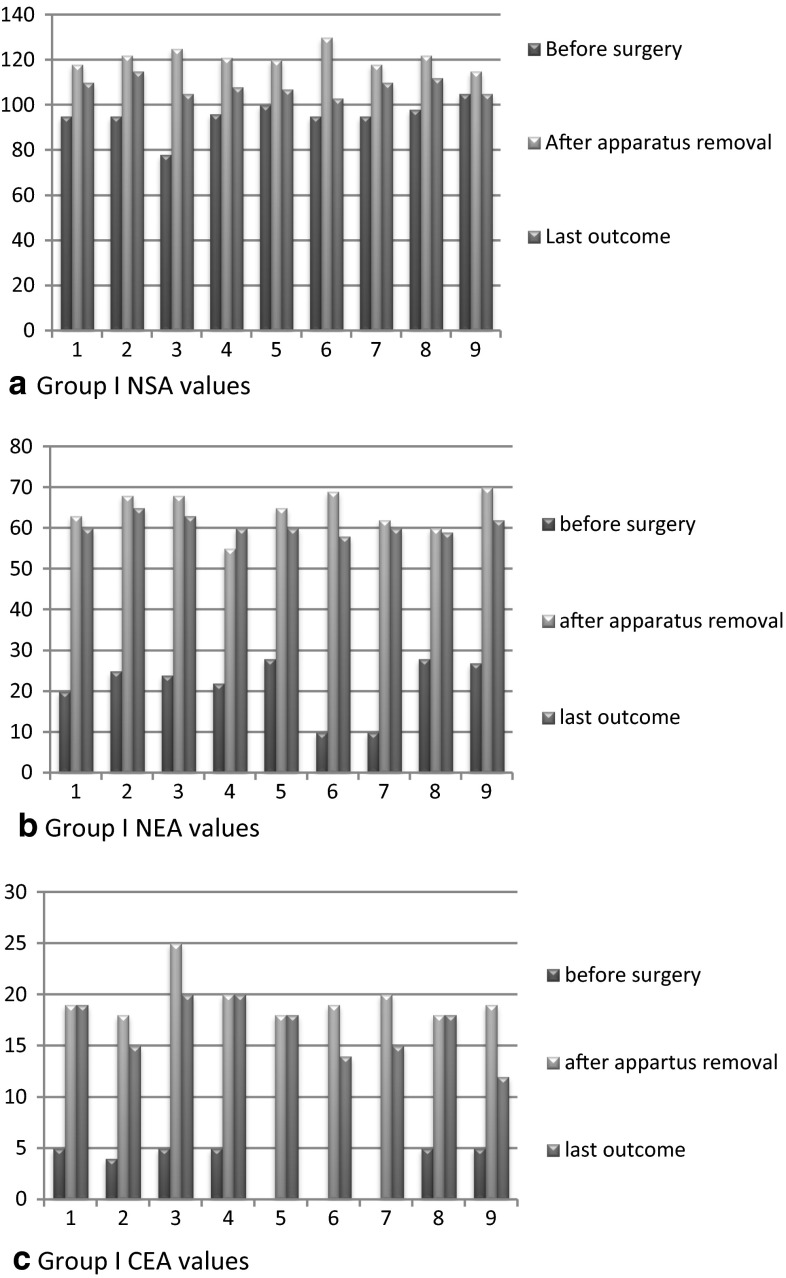
Diagrammatic representation of preoperative, after apparatus removal, and last outcome of group I NSA, NEA, and CEA values

**Fig. 5:**
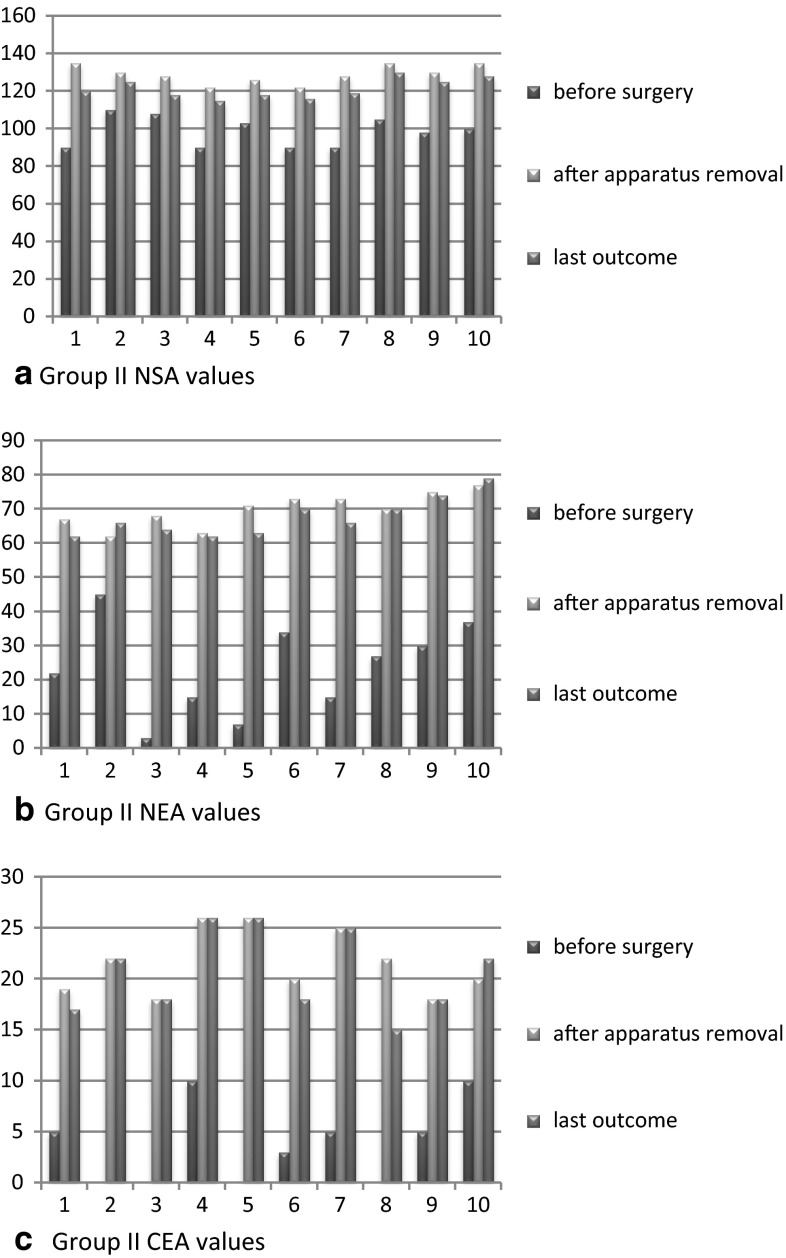
Diagrammatic representation of preoperative, after apparatus removal, and last outcome of group II NSA, NEA, and CEA values

Group I included nine children aged 4–8 years (mean 6.66 years), while group II included ten patients aged 9–15 years (mean 11.8 years). Clinical features in the two analyzed groups of patients are limping, positive Trendelenburg sign, limitation of abduction, shortening of the limb, and moderate pain, which was observed more in patients of group II. Radiographic signs included valgus deviation of the epiphysis, reducing NSA, shortening of the neck, high location of the tip of the greater trochanter, and subluxation of the femoral head. The average gain in neck length was 1.77 cm for group I (range 1–3 cm) and 3 cm for group II (range 1–4 cm). Postoperatively, significant improvement in average radiographic parameters was observed in both groups. For group I, the preoperative mean NSA was 95 ± 2.9, with postoperative significant change (121 ± 1.7; *p* < 0.01). However, at final assessment, it showed partial recurrence (108 ± 1.4; *p* < 0.01). The preoperative mean NEA was 21.5 ± 3.9, with postoperative significant change (64 ± 0.9; *p* < 0.001). The preoperative mean ATD (mm) was −8.4 ± 2.5, with postoperative significant change (14.5 ± 0.9; *p* < 0.001) and partial recurrence at final assessment (8 ± 1.04; *p* < 0.01). The preoperative mean CEA was 3 ± 0.9, with postoperative significant change (19.5 ± 0.8; *p* < 0.001). For group II, the preoperative mean NSA was 98 ± 2.5, with postoperative significant change (129 ± 1.6; *p* < 0.001). However, at final assessment, it showed partial recurrence (121 ± 1.6; *p* < 0.01). The preoperative mean NEA was 23.5 ± 4.3, with postoperative significant change (70 ± 1.6; *p* < 0.001). The preoperative mean ATD (mm) was −13.4 ± 3.1, with postoperative significant change (17.4 ± 0.8; *p* < 0.001). The preoperative mean CEA was 3.8 ± 1.2, with postoperative significant change (21.6 ± 1.0; *p* < 0.001) (Table 3; Figs. 4 and 5).

**Fig. 6:**
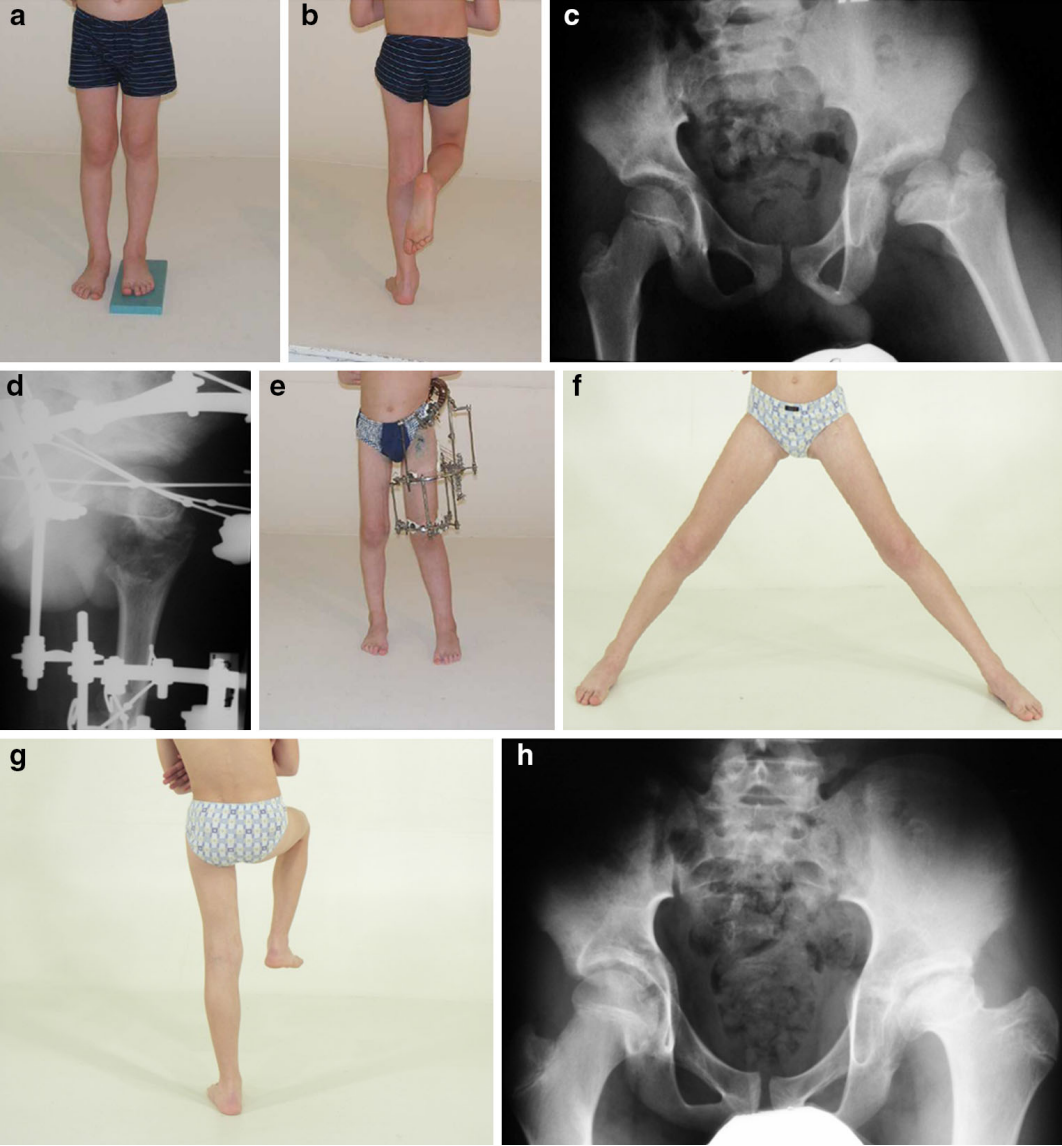
**a**, **b** Preoperative left leg shortening and positive Trendelenburg sign in a 6-year-old boy with septic coxitis sequelae, Kalamchi II. **c** Preoperative AP pelvic radiograph with characteristic left proximal femoral ischemic deformities. **d** Neck lengthening with regenerate formation. **e** Patient with apparatus during the consolidation phase. **f**, **g** Three years postoperative abduction and negative Trendelenburg sign. **h** AP radiograph 5 years postoperatively showing remodeling of the left proximal femur

The functional results of group I patients (Fig. 6) in accordance with Colton’s criteria are six good (13–14 points) and three satisfactory (11–12 points). The radiological findings of group I patients in accordance with Kruczynski’s criteria are four good patients, four satisfactory, and one unsatisfactory.

**Fig. 7:**
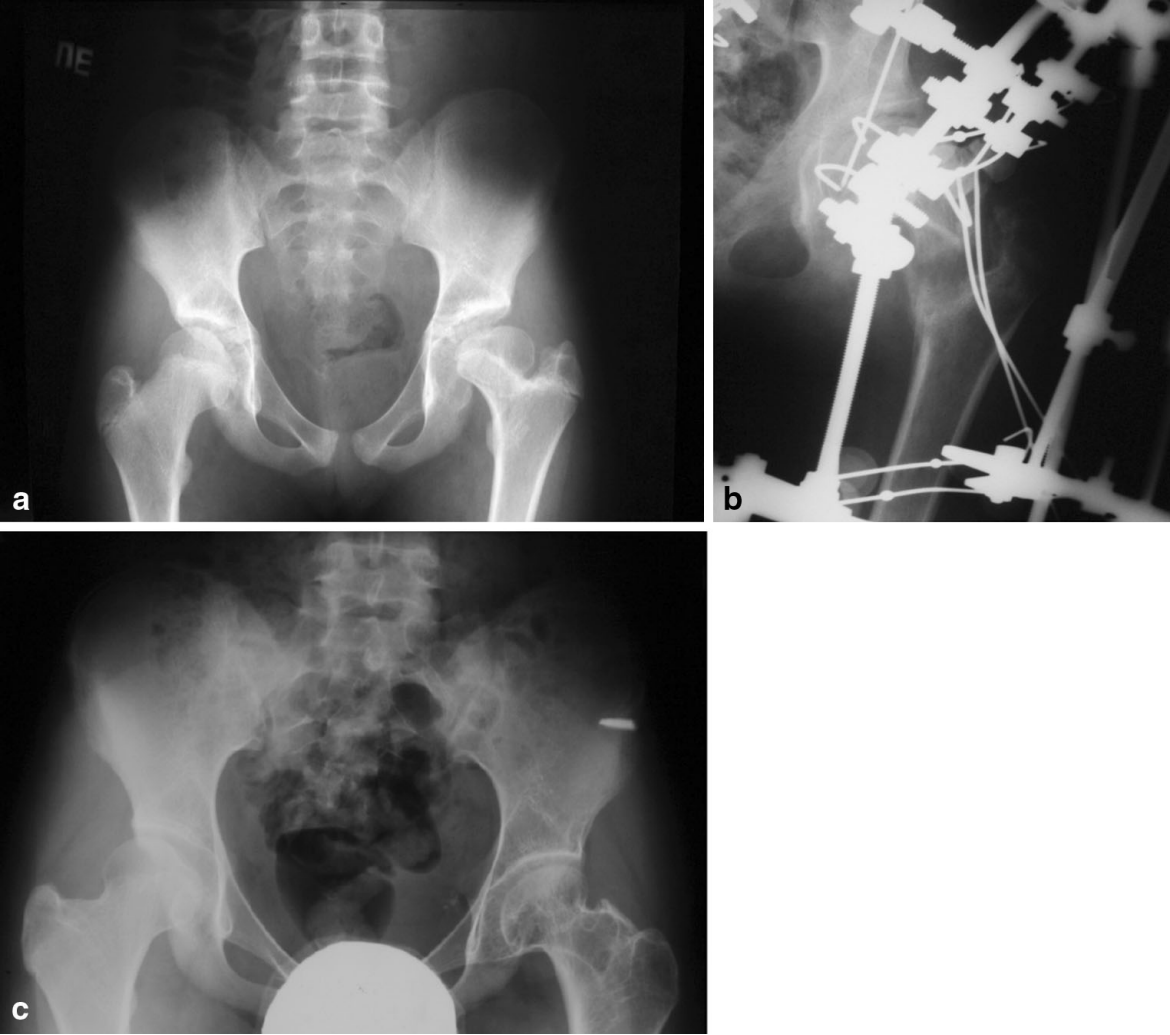
**a** A 13-year-old girl with *left-side* DDH deformity Kalamchi IV with caput valgum, neck shortening, and proximally located hyperplastic greater trochanter. **b** Distraction femoral neck lengthening after pertrochanteric osteotomy. **c** Three years after treatment, with a 3-cm gain in neck length and correction of deformities

The functional results of group II patients (Fig. 7) in accordance with Colton’s criteria are two very good (15–16 points), seven good (13–14 points), and one satisfactory (11–12 points). The radiological findings of group II patients in accordance with Kruczynski’s criteria are seven good and three satisfactory.

Observed complications included shortening of 1 cm in three patients from group I, three superficial wire infections, and one deep infection in both groups. Aseptic necrosis of the femoral head occurred in a female patient aged 8 years old in group I with DDH 3 months after device removal. The cause was early loading on the operated limb with resulting neck–shaft varus deformation and subluxation of the femoral head. Retarded consolidation in the distraction area was observed in a male patient aged 9 years old in group II with aseptic necrosis and type IV Kalamchi classification. This lead to an increase in the duration of fixation of an additional 5 months.

## Discussion

Avascular necrosis of the femoral head can result from many causes that interfere with its blood supply. Reduction manipulation is a leading cause following DDH treatment [16]. Deformities include coxa vara, caput valgum resulting from growth disturbance to the lateral femoral physis [1], femoral neck shortening, and proximal migration of the greater trochanter with hyperplasia and impingement on the acetabular rim with abduction. The resulting disturbed biomechanics accentuates hip joint deformation and ischemia in a vicious circle manner and, eventually, early osteoarthritic changes. Many proximal femoral osteotomies have been described with different results. This included single, double, and triple [7–12] osteotomies. Buess and Morscher [9] used a technique with lengthening of the femoral neck and distal transfer of the greater trochanter. They used three osteotomies, at the greater trochanter, one proximal, and the third an oblique osteotomy at the level of the distal femoral neck. In 15 patients with 16 operated hip joints, the results were satisfactory in 14 of the 16 hips. They recommend this technique to be performed in young patients with little or no degenerative changes.

 Sokolovsky and Sokolovsky [11] carried out an intertrochanteric osteotomy with posterior rotation of the femoral head and neck. There were 45 hips aged from 6 to 18 years. In ten patients, femoral osteotomy was combined with a variety of pelvic procedures. After a mean follow-up of 5 years, they had excellent results in nine patients, good in 17, fair in seven, and poor in four. Garrido et al. [18] described a limited technique for distal and lateral transfer of the greater trochanter. Hasler and Morscher [5] described two parallel osteotomies of the femur at the upper and lower border of the femoral neck, followed by distalization of the greater trochanter into a normalized position. Lengsfeld et al. [19] described a valgus osteotomy technique to lengthen the neck. Among 24 patients, they had good or satisfactory results in 23 cases. None of these techniques address all the deformities simultaneously as compared to our technique.

 The role of proximal femoral and acetabular osteotomies has been studied by Ganz et al. [20]. The study provided an algorithm for the treatment of complex hip deformities. The role of additional proximal femoral osteotomy for adequate coverage and containment of the femoral head is emphasized even in patients with established hip arthritis. A more comparable osteotomy to our technique was first described by Nishio and Sugioka [21], who performed a C-shaped varus intertrochanteric osteotomy to address avascular necrosis of the femoral head. Later, Sakano et al. [22] studied the long-term outcome of this technique on 20 hips and found excellent results, with 18 hips survived without collapse after a mean follow-up of 48 months. Although the technique eliminates valgus deviation of the head, the position of the greater trochanter is reduced but not normalized. Another difficulty is that the angle of rotation is unpredictable preoperatively and shortening of the limb is not corrected [22]. In contrast, the distal distraction and neck control obtained in our technique enabled for bringing the greater trochanter into a comparable level with the normal side, predicting the angle of anteversion, and restoring limb length. Another comparable technique performed by Papavasiliou et al. [23] provides restoration of the relationships between the head and the tip of the greater trochanter, increasing the neck length, and compensating for shortening of the limb. However, the correction degree of caput valgus is limited by the large size of the L-shaped proximal femoral osteotomy fragment. In contrast, our technique enabled correction of caput valgus deformity to a greater degree that is comparable with the normal side.

The application of the Ilizarov apparatus achieved simultaneous correction of the deformities and lengthening of the neck with a controlled rate of length gained. This technique has the characteristic of low-energy percutaneous osteotomy and gradual distraction which is known for promoting angiogenesis and new bone cells formation [14, 24]. In addition, the versatility the apparatus in adjusting the degree of correction of bone deformities is unlikely to be obtained when using fixed devices, where the amount of correction and length gained are set by the end of the operation. The procedure is also minimally invasive, with less blood loss and does not need surgical removal of the device.

With regard to our results, most of the radiographic values excluding the LT are consistent with the boundary of age norms. Examination of the children in the first group revealed partial recurrence of the deformity of the proximal femur at last outcome. This can be observed by the decrease in the values of the NSA, ATD, and LT. Nevertheless, the orientation of the head relative to the neck and the head containment within the acetabulum did not change significantly. We think that this is expected, as the child is growing and later correction can be performed. However, the recurrence is confined to the neck–shaft varus and in the ATD, as there is no observation of significant change of the NEA or recurrence of caput valgum deformity in the last outcome assessment. The correction of neck varus can be done with simple corrective osteotomy. The decrease of the ATD can be addressed by trochanteric advancement osteotomy if hip abductors function is affected. Joo et al. [25] studied the result of this surgery in Perthes patients with relative overgrowth of the greater trochanter and found that it doesn’t always improve hip function, so we recommend that the decision of revision surgery to be taken when clinical and radiological aspects are assessed together. Group II patients showed a significant reduction only in the average NSA, which corresponded to the lower limit of normal values. This change is unlikely to be increased, as this group is reaching skeletal maturity. In both groups, no significant change was observed for recurrence of caput valgum or change in head containment represented by postoperative sustained values of NEA and CEA, respectively.

Complications included pin tract infection I and II degrees in accordance with Paley’s criteria [26]. This was treated by changing dressing and oral antibiotics. Maintained shortening of 1 cm in three patients was considered for conservative observation. The retarded consolidation was addressed by increasing the duration of fixation. The case with aseptic necrosis was missed to follow-up after one and a half years. We insist that clear postoperative care instructions should be given to the patients during hospital stay and after discharge to minimize the risk of complications, especially those related to weight bearing. We recommend that this technique be performed by pediatric orthopedic surgeons experienced with the Ilizarov technique. A larger number of patients and longer follow-up terms are needed in order to further verify the application of this method.

## Conclusion

Overall, the results of the clinical and radiographic evaluations showed a sufficient proportion of good results (58.9 %), with restoration of the anatomical structures and their relations. We believe that the results are encouraging for application of this method, with enhanced biological environment we provide an additional technique to manage proximal femoral ischemic deformities in pediatric patients.
